# Comprehensive *ex vivo* and *in vivo* preclinical evaluation of novel chemo enzymatic decellularized peripheral nerve allografts

**DOI:** 10.3389/fbioe.2023.1162684

**Published:** 2023-03-30

**Authors:** Óscar Darío García-García, Marwa El Soury, Fernando Campos, David Sánchez-Porras, Stefano Geuna, Miguel Alaminos, Giovanna Gambarotta, Jesús Chato-Astrain, Stefania Raimondo, Víctor Carriel

**Affiliations:** ^1^ Tissue Engineering Group, Department of Histology, University of Granada and Instituto de Investigación Biosanitaria ibs.GRANADA, Granada, Spain; ^2^ Doctoral Program in Biomedicine, University of Granada, Granada, Spain; ^3^ Department of Clinical and Biological Sciences and Neuroscience Institute Cavalieri Ottolenghi (NICO), University of Torino, Orbassano, Italy

**Keywords:** acellular graft, decellularization, decellularized nerve allograft, peripheral nerve repair, tissue engineering

## Abstract

As a reliable alternative to autografts, decellularized peripheral nerve allografts (DPNAs) should mimic the complex microstructure of native nerves and be immunogenically compatible. Nevertheless, there is a current lack of decellularization methods able to remove peripheral nerve cells without significantly altering the nerve extracellular matrix (ECM). The aims of this study are firstly to characterize *ex vivo*, in a histological, biochemical, biomechanical and ultrastructural way, three novel chemical-enzymatic decellularization protocols (P1, P2 and P3) in rat sciatic nerves and compared with the Sondell classic decellularization method and then, to select the most promising DPNAs to be tested *in vivo*. All the DPNAs generated present an efficient removal of the cellular material and myelin, while preserving the laminin and collagen network of the ECM (except P3) and were free from any significant alterations in the biomechanical parameters and biocompatibility properties. Then, P1 and P2 were selected to evaluate their regenerative effectivity and were compared with Sondell and autograft techniques in an *in vivo* model of sciatic defect with a 10-mm gap, after 15 weeks of follow-up. All study groups showed a partial motor and sensory recovery that were in correlation with the histological, histomorphometrical and ultrastructural analyses of nerve regeneration, being P2 the protocol showing the most similar results to the autograft control group.

## Graphical Abstract

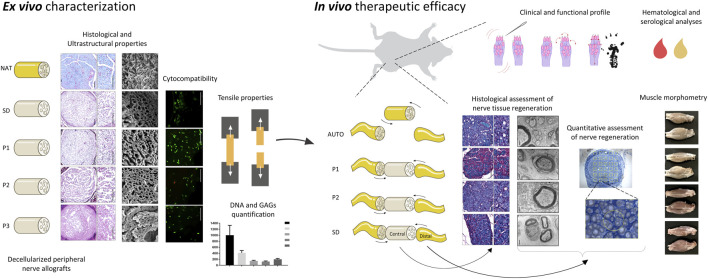

## 1 Introduction

Peripheral nerves (PNs) are highly specialized organs that connect the central nervous system through motor, sensory or autonomic pathways, with distal target organs (Carriel et al., 2014). Histologically, PNs are composed to two well-defined tissues, the parenchyma or functional unit, formed by peripheral nerve fibers (PNFs), and the stroma or structural unit, formed by three specialized layers of connective tissue (the epineurium, the perineurium and the endoneurium) that provide blood and lymphatic vessels, and confer the structural and biomechanical properties to these essential organs (Geuna et al., 2009; Hromada et al., 2022). Due to their wide anatomical distribution PNs are often affected by traumatic injuries of diverse nature or iatrogenic injuries during surgical procedures (Robinson, 2000). In severe structural PN injuries (PNI), the surgical implantation of a graft is needed to re-establish the nerve continuity, which is crucial to promote the nerve tissue regeneration and reinnervation of distal organs, such as muscle fibers (Carriel et al., 2014). Nowadays, nerve autograft remains the gold standard technique to repair PNIs up to approximately 5 cm in length. However, it has some well-documented disadvantages (Carriel et al., 2014; Yi et al., 2019) and thus new therapeutic alternatives are still needed (Lovati et al., 2018).

In order to overcome the therapeutic needs in PN repair, a wide range of bioengineered conduits or neural substitutes have been generated and characterized by tissue engineering (TE) (Carriel et al., 2014). This vast research has revealed that in order to promote nerve tissue regeneration these substitutes must meet certain criteria such as an adequate biodegradability rate, porosity, 3D organization and alignment, pro-regenerative chemical composition, and physicochemical properties. All these factors are crucial to stimulate Schwann cells (SCs) at the injury site to support and guide the complex axonal regeneration process (Gedeon et al., 2019). In this context, different natural or synthetic biomaterials were used to create nerve substitutes by TE highlighting some fabrication techniques, for example, dip-molding (Ao et al., 2006; Wang and Huang, 2008; Haastert-Talini et al., 2013), soft intraluminal fillers (Carriel et al., 2013; Carriel et al., 2017b; Rayner et al., 2021), physical film rolling (Carriel et al., 2017c; Chato-Astrain et al., 2018), solvent casting-salt leaching (Guo et al., 2012), electrospinning (Pozzobon et al., 2021), crosslinking (Agarwal et al., 2021), braiding and bioprinting (Tao et al., 2020). Over the last years, different bioengineered models showed highly promising experimental results, but they remain less efficient than the autograft technique (Houshyar et al., 2019). These differences were related to the highly complex nerve microstructure and extracellular matrix (ECM) composition, which was not accurately achieved within the bioengineered substitutes and thus the generation of nerve substitutes with these microstructural features remains a challenge.

In this context, the generation of decellularized peripheral nerve allografts (DPNAs) emerged as a promising alternative in the field. These tissue-specific 3D natural matrices maintain the complex 3D nerve microarchitecture, the ECM biomolecular composition and the biomechanical integrity of these organs minimizing their immunogenicity (Gilbert et al., 2006; Kneib et al., 2012). Moreover DPNAs have several advantages over other tissue-engineered strategies as they can be immediately available for use, they are less expensive than other nerve substitutes, and their use is supported by a growing preclinical and even clinical evidence (Carriel et al., 2014; Lovati et al., 2018; Contreras et al., 2022; Roballo et al., 2022). However, there is variability in the efficacy of the decellularization methods used and currently an ideal procedure does not exist yet. Although most methods available may efficiently remove the cellular content, it is still necessary to develop novel decellularization methods to ensure an adequate preservation of the 3D structure and molecular composition of the obtained ECM. In previous studies, we generated, by using different methods, novel DPNAs with different degrees of decellularization, ECM preservation and biomechanical behavior. Once tested *in vivo*, promising results were obtained, but clear improvements are needed to superate the efficacy of nerve autograft (Keane et al., 2015; Philips et al., 2018b). For this reason, and in line with our previous works, three novel combined or chemo-enzymatic decellularization procedures were designed, described, and comprehensively characterized *ex vivo* at the histological, ultrastructural, biomechanical, and biological level. Based on the *ex vivo* characterization, two DPNAs were selected, and their therapeutic efficacy was determined in the repair of 10-mm sciatic nerve gap in Wistar rats.

## 2 Material and methods

### 2.1 *Ex vivo* characterization of DPNAs

#### 2.1.1 Laboratory animals

In this study 12 weeks old Wistar rats were used. Animals were kept during the whole study in the Experimental Unit of the University Hospital Virgen de las Nieves in Granada (Spain). Rats were housed in clean rooms with 12-h light/dark cycle, temperature-controlled environment (21°C ± 1°C), and with *ad libitum* access to standard rat chow and tap water. All procedures included in this study were conducted according to the Spanish and European regulations for animal experimentation (EU directive No. 63/2010, RD 53/2013), comply with the Animal Research: Reporting of *In vivo* Experiments (ARRIVE) guidelines and were approved by Ethics and Animal Experimentation Committee of Granada University approval No. 29/03/2022/052, Grant FIS P20-0318.

#### 2.1.2 Nerve isolation and chemical decellularization

Sciatic nerves were harvested from 35 adult Wistar rats 12 weeks old. To obtain the sciatic nerves, the animals were deeply anaesthetized by an intraperitoneal injection of acepromazine (Calmo-Neosan^®^, Boehringer Ingelheim Animal Health España, S.A.U., Barcelona, Spain, 0.001 mg/g body weight), ketamine (Imalgene 1,000^®^, Merial, Lyon, France, 0.15 mg/g body weight) and atropine (Pfizer, New York, NY, United States, 0.05 μg/g body weight) and then euthanized by an intraperitoneal injection of Eutanex^®^ solution (0.5 mL/animal). Subsequently, ∼3 cm of both sciatic nerves (*n* = 70 nerves) were removed and cryopreserved (10% DMSO in fetal bovine serum at −80°C) until use as previously described (Chato-Astrain et al., 2020b; El Soury et al., 2021; Garcia-Garcia et al., 2021; Contreras et al., 2022).

A total of 60 PNs were defrosted at room temperature (RT), washed in distilled water, sectioned into fragments 1 cm in length (except those used in biomechanical test ∼3 cm) and distributed randomly to the different decellularization groups. In this study, 3 novel decellularization protocols (called P1, P2 and P3) were developed, characterized and compared with the well-known Sondell decellularization method (SD) (Sondell et al., 1998) and native nerves (NAT).

Decellularization procedures have several steps and each one has a duration of 24 h unless specified. In addition, after each step, the samples were washed three times with distilled water. Please note that all reagents used were purchased from Merck (Darmstadt, Germany). The main steps of each protocol were as follow:

- Decellularization protocol 1 (P1): Distilled water, 0.1% sodium dodecyl sulphate (SDS cat n° L3771), 0.6% Triton X-100 (cat no T8787, 1% sodium deoxycholate (SDC, cat no D6750) and 2 × consecutive treatments of 1 h enzymatic mix solution 100 mg/L DNase (DN25) and 20 mg/L, RNase (cat no R4875) in 0.1 M phosphate buffered saline (PBS).
- Decellularization protocol 2 (P2): Distilled water, 1% SDS, 3% Triton X-100, 4% SDC and 2 h enzymatic mix solution.
- Decellularization protocol 3 (P3): Same procedure that P2 replacing enzymatic mix solution for 4 h of 3% peracetic acid (PAA).
- SD control group: Two cycles of 3% Triton X-100% and 4% SDC alternatively, 24 h each step.

Once decellularization was complete, DPNAs, from each procedure, were abundantly washed in distilled water and kept in PBS at 4°C until further use. The whole procedure was conducted at RT and using constant agitation (24 rpm), except the enzymatic digestion which was conducted at 37°C. Native nerves were unfrozen to be used as control group.

#### 2.1.3 Histological and ultrastructural characterization of DPNAs

The efficacy of the decellularization process as well as the degree of ECM preservation following nerve decellularization was assessed by a combination of light and electron microscopy methods following previous recommendations (Philips et al., 2018b). Native nerves and each generated DPNAs (*n* = 3) were fixed for 48 h in 10% neutral buffered formaldehyde solution at RT, dehydrated and transversally embedded in paraffin for light microscopy (Carriel et al., 2017a). In case of samples for electron microscopy, they were fixed in 2.5% glutaraldehyde (in 0.05 M cacodylate buffer (pH 7.2) at 4°C overnight), washed in cacodylate buffer, post-fixed in 2% OsO_4_ (1 h) and included in Glauerts resin mixture as previously described (Ronchi et al., 2014; El Soury et al., 2021; Garcia-Garcia et al., 2021). The light and electron microscopy methods used in this study were as follow:

- General histology and cell removal was evaluated by using hematoxylin and eosin (HE) and the intercalant fluorochrome 4′,6-diamidino-2-phenylindole (DAPI) respectively.
- In order to evaluate the removal of cells main components, the presence of neurofilament (NFL, axons, RRID:AB_477262), vimentin (VIM, stromal and Schwann cells, RRID:AB_477627) and S-100 protein (Schwann cells, RRID:AB_10013383) were evaluated by immunohistochemistry.
- The structure and preservation of the ECM was evaluated by using Alcian Blue (AB, proteoglycans) and Picrosirius (PS, fibrillar collagens) histochemical methods whereas the basal-membrane glycoprotein laminin (LAM, RRID:AB_298179) was determined by immunohistochemistry.
- To evaluate the myelin sheath remnants and fibrillar collagen network, sections were stained with MCOLL histochemical technique as previously described (Carriel et al., 2011; Garcia-Garcia et al., 2023).
- The ultrastructural features of the generated DPNAs were evaluated by scanning and transmission electron microscopy (SEM and TEM respectively).

The antibodies used and technical details of all immunohistochemical procedures are summarized in the Supplementary Table S1.

#### 2.1.4 Biochemical analysis of DNA and glycosaminoglycans content

Total DNA was extracted and purified from tissue samples using the QIAamp DNA Mini Kit (Quiagen, Hilden, Germany) following the manufacturer’s recommendations. Purified DNA was quantified with a NanoDrop 2000 spectrophotometer (Thermo Fisher Scientific, Waltham, United States) and five technical measures were taken for each tested sample. For quantification of sulfated glycosaminoglycans (sGAGs), the Blyscan Sulfated Glycosaminoglycan Assay (Biocolor, Carrickfergus, United Kingdom) was used according to the manufacturer protocol. The colorimetric reaction was analyzed with a spectrophotometer (ASYS UVM340) and DigiRead software (Biocrom Ltd., Cambridge, United Kingdom) at 656 nm (maximum peak). DNA and sGAGs values were normalized with the dry weight of the tissue samples before extraction (*n* = 5 each technique).

#### 2.1.5 Biomechanical response of DPNAs under tensile test

For the purpose of assessing the biomechanical properties of the different generated DPNAs, a tensile test was performed by using an Instron 5943 (Instron, Needham, United States) as previously described (Carriel et al., 2017c; Philips et al., 2018a; Garcia-Garcia et al., 2021). Briefly, four samples of each condition (segments of ∼3 cm length) were placed between the instrument holders leaving a constant distance of 1 cm. Subsequently, the stress, strain, and extension at fracture as well as the Young Modulus values were calculated. Tensile tests were run within hydrated samples at a constant strain rate of 10 mm/min and a pre-charge value of 5·10^−3^ N at RT.

#### 2.1.6 *Ex-Vivo* cytocompatibility

To assess the biocompatibility of generated DPNAs, the interaction of rat adipose-derived mesenchymal stem cells (ADMSC) with these acellular matrices was evaluated as previously described (Garcia-Garcia et al., 2021). Briefly, DPNA segments of 0.5 cm were longitudinally opened and placed in 3.5% (w/v) type I agarose (Merck, Steinheim, Germany) pre-coated Costar^®^ 24-well cell culture plate surface (Corning, New York, United States), and then 2 × 10^4^ ADMSCs (from passage VII (stemness profile characterized by immunohistochemistry and cytometry expressing positivity in CD90 = 96.27% and CD29 = 98.72% and negativity in CD45 = 98.87% markers as previously described (Sun et al., 2011; Lotfy et al., 2014)) were seeded in the inner surface of each sample corresponding to the endoneurial compartment. Subsequently, wells were supplemented with basal culture medium [Dulbecco’s modified Eagle medium (DMEM) supplemented with 10% fetal bovine serum (FBS) and 1% antibiotic and antimycotic solution 100x (all products from Merck, Steinheim, Germany)] and cultured for 48 h under standard conditions (37°C and 5% CO_2_). Finally, the cell-biomaterial interactions were determined by using Live/Dead^®^ Cell Viability Assay (L/D) (Thermo-Fisher Scientific, Portland, United States) and Water-soluble tetrazolium-1 (WST-1) assay (Roche, Mannheim, Germany) as previously recommended (Philips et al., 2018b). L/D and WST-1 commercial kits were performed following the manufacturer’s recommendation and previous studies (El Soury et al., 2021; Garcia-Garcia et al., 2021). L/D results were analyzed with a Nikon Eclipse Ti fluorescence microscope equipped with a Nikon DXM 1200c Digital Camera (Nikon, Tokyo, Japan) showing viable and metabolically active cells in green color, while dead cells allowed the intercalant agent ethidium to enter to the nucleus emitting red fluorescence. The colorimetric reaction obtained with WST-1, corresponding to the cellular metabolic activity of the ADMSCs seeded on the DPNAs generated, was measured with a spectrophotometer (ASYS UVM340) and DigiRead software (Biocrom Ltd., Cambridge, United Kingdom) at 450 nm (maximum peak).

In all assays, non-cell seeded-DPNAs were placed in agarose pre-coated wells and maintained in the same culture condition and used as a negative control. Additionally, 2 × 10^4^ ADMSCs were seeded in wells without agarose-coating and used as 2D positive or negative technical controls. In the case of the 2D negative control, an irreversible cell-membrane and nuclei damage was induced by using 2% Triton X-100. Finally, all these assays were performed in quintuplicate.

### 2.2 *In vivo* evaluation of DPNAs

In this study, the DPNAs obtained with P1 and P2 showed optimal *ex vivo* properties to be tested *in vivo* in the rat model of sciatic nerve injury and repair.

#### 2.2.1 Surgical procedure and *in vivo* experimental groups

A total of 24 male adult Wistar rats were deeply anesthetized, as described in Section 2.1.2 and then a segment of 1 cm of left sciatic nerve was carefully harvested from each animal (Chato-Astrain et al., 2018; Chato-Astrain et al., 2020b). Afterward, the animals were randomly assigned (*n* = 6 per group) to be repaired through the microsurgical implantation of DPNAs (P1, P2 and SD groups) and autograft technique (AUTO). SD and AUTO groups were used as positive controls of nerve tissue regeneration (*n* = 6 in each). In AUTO group, the removed nerve segments were rotated in 180° and reimplanted. In addition, in each operated animal the right leg was kept as healthy control and three independent healthy animals were also kept during the whole experiment and used as healthy control group (CTR).

#### 2.2.2 Clinical assessment

Operated and healthy animals were subjected to clinical, sensory, and motor function recovery evaluation. Clinically, animals were subjected to sensory and motor function recovery analyses after 15 weeks of nerve repair. These analyses were conducted following previous described and recommended procedures (Vleggeert-Lankamp, 2007; Lovati et al., 2018; Chato-Astrain et al., 2020b).

##### 2.2.2.1 Sensory recovery assessment

The pinch test was performed applying a mild pinching stimulus to the skin of the left hindlimb with forceps, from the toe to the knee joint, until a withdrawal reaction was observed. Response was graded from 0 to 3: 0 = no withdrawal response, 1 = response to stimulus above the ankle, 2 = response to stimulation distal to the ankle in the heel/plantar region, and 3 = response to stimulation in the metatarsal region (Carriel et al., 2013; Chato-Astrain et al., 2020b).

##### 2.2.2.2 Motor functional recovery assessment

Sciatic functional index (SFI) test was performed through the evaluation of the walking track as described previously (Monte-Raso et al., 2008). Briefly, both feet plantar regions were stained with blue ink, and animals were introduced on a Plexiglas^®^ device (1-m length, 10-cm width, and 15-cm height) covered with white paper (Carriel et al., 2013; Chato-Astrain et al., 2018; Chato-Astrain et al., 2020b) where the footprints were recorded. In the footprints, the following parameters were measured 1) the print length (PL), which is the distance from the heel to the third toe; 2) the toe spread (TS), which is the distance from the first to the fourth toe; and 3) the intermediary toe spread (ITS), which is the distance from the second to the fourth toe. These data were used to calculate the SFI with the following formula: SFI = −38.3 × PL + 109.5 × TS + 13.3 × ITS− 8.8. It should be considered that it was not possible to determine the SFI in 5 animals [AUTO group 1), SD group 2), P1 and P2 groups (1 each)].

For the toe-spread test, an indicator of motor recovery, animals were suspended by the tail, and the abduction and extension reaction of the toes was evaluated. Results were scored from 0 to 3: 0 = no toe movement, 1 = some sign of toe movement, 2 = toe abduction, and 3 = toe abduction with extension (Carriel et al., 2013; Chato-Astrain et al., 2018).

#### 2.2.3 *Postmortem* studies

Once the clinical and functional assessments were performed, animals were deeply anesthetized, and 1.5 mL of blood was collected for hematological and serological analyses. Then, animals were euthanized and both sciatic nerves (operated and healthy) and both hindlimbs were harvested for further analysis. Please note that details about anaesthesia and euthanasia procedures were described in Section 2.1.2. Nerves were used for histological and morphometric analyses, whereas hindlimbs were used to determine changes in the muscle weight.

##### 2.2.3.1 Hematological and serological analyses

For these analyses, 1.5 mL of blood from each animal was collected in heparinized Eppendorf and subjected to hematological and serological analyses as previously described (Chato-Astrain et al., 2020b; Campos et al., 2021). Briefly, hematological counting analysis was performed with a Sysmex KX-21 N automatic analyzer (Florida, United States). For serological analyses, the serum was obtained by the centrifugation (3,500 rpm for 15 min) and the serum was analyzed with a Cobas c311 clinical chemistry analyzer and biochemical kits following the manufacturer recommendations (Roche Laboratories). The hematological and serological parameters analyzed were red blood cell count (RBC), hemoglobin (HGB), mean corpuscular volume (MCV), white blood cell count (WBC), lymphocytes (LYM), mixed cell count (MXD), neutrophils (NEUT), and mean platelet volume (MPV). The biochemical parameters evaluated were alanine transaminase (ALT), aspartate aminotransferase (AST), total bilirubin (BILT), creatinine (CRE), urea (UREA), low-density lipoprotein cholesterol (LDLC), high-density lipoprotein cholesterol (HDLC), triglycerides (TRIGL), amylase (AMYL). In addition, when it was possible, values obtained were compared with the hematological standard range described in the study of Kampfmann et al. (2012).

##### 2.2.3.2 Muscle morphometry

The hindlimbs were obtained by disarticulating the knee and ankle, without damaging the muscles attached to the tibia and fibula. The legs were fixed during 24 h in 10% formaldehyde, washed in tap water and then subjected to wet weight calculation. Additionally, the gastrocnemius and tibialis anterior muscles were carefully dissected from both limbs (healthy and operated legs) and then muscle wet weight was measured independently. The percentage of weight loss was calculated in each animal as {[(lesion side muscle/contralateral side)*100]−100} as described previously (Shea et al., 2014; Chato-Astrain et al., 2018; Chato-Astrain et al., 2020b).

##### 2.2.3.3 Histological assessment of nerve tissue regeneration

Harvested healthy and operated nerves (*n* = 6) were processed for conventional histology, as described above, whereas the distal nerve stump was used for quantitative ultrastructural analysis. For conventional histology, central portion of the implanted grafts were selected and stained with histochemical and immunohistochemical methods as recommended (Chato-Astrain et al., 2020a; Garcia-Garcia et al., 2023). General morphology was evaluated by HE, and the degree of myelination and collagen organization pattern were assessed by MCOLL. Furthermore, PN regeneration was confirmed by indirect immunohistochemistry for S-100 (Schwann cells, RRID:AB_10013383), Grow associated protein 43 (GAP-43, immature axons RRID:AB_1310252) and neurofilament (NFL, regenerated mature axons, RRID:AB_477262). The technical details of the immunohistochemical procedures and antibodies used are summarized in Supplementary Table S1.

##### 2.2.3.4 Quantitative and ultrastructural assessment of nerve regeneration

Distal nerve stump (adjacent to distal portion of each graft) was harvested and processed for transmission electron microscopy as described in Section 2.1.3 *Histological and ultrastructural characterization of DPNA.*

For quantitative morphometry analysis, semithin transversal sections of 2.5 µm thickness were obtained and stained with 1% toluidine blue staining for high resolution light microscopic examination and design-based stereology using DM4000B microscope equipped with a DFC320 digital camera (Leica Microsystems). Images were processed with ImageJ software (version 1.53 k, National Institute of Health, Bethesda, MD, United States). In each section, the total cross-sectional area of the whole nerve was measured at the light microscopic level and 15–25 sampling fields were selected using a systematic random sampling protocol (Geuna et al., 2000; Ronchi et al., 2014). In each sampling field, a two dimensional dissector procedure, which is based on sampling the “tops” of fibers, was adopted in order to avoid the “edge effect” (Geuna et al., 2000). Mean fiber density was then calculated by dividing the total number of nerve fibers within the sampling field by its area (N/mm^2^). Total fiber number (N) was finally estimated by multiplying the mean fiber density by the total cross-sectional area of the nerve. Moreover, both fiber and axon area were measured, and the diameter of fiber (D) and axon (d) were calculated. These data were used to calculate myelin thickness [(D−d)/2], the g-ratio (D/d). These analyses were conducted in triplicate.

Ultrastructural assessment of nerve regeneration was performed selecting 3 random samples of each group and obtaining representative images of each condition*.*

## 3 Results

### 3.1 *Ex vivo* characterization of novel DPNAs

In this study, three novel decellularization protocols (P1, P2 and P3) were developed, characterized, and compared with the classic decellularization method of Sondell (SD) (Sondell et al., 1998) and native nerves (NAT) were used as control group. These decellularization methods were described in the Experimental section.

#### 3.1.1 Histological and ultrastructural properties of the DPNAs

**FIGURE 1:**
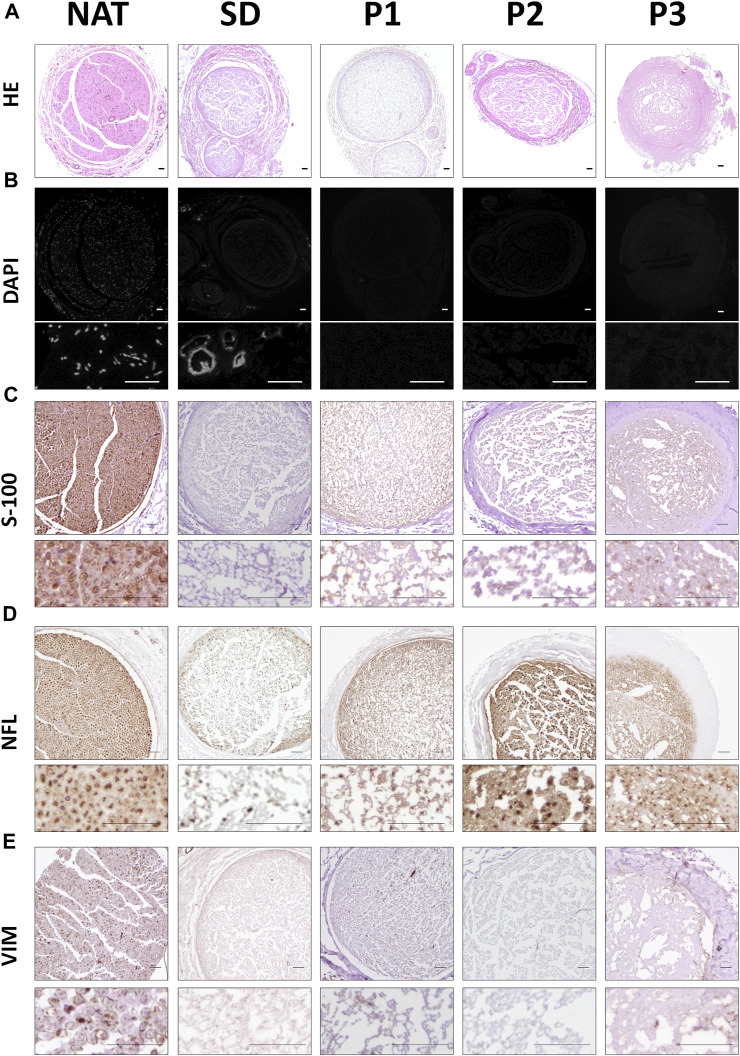
Histological, histochemical and immunohistochemical evaluation of cellular components remained in the different DPNA groups. **(A)** General morphology of the study groups was assessed by hematoxylin-eosin histochemical method. **(B)** Fluorescent microscopy images of nerves stained with the intercalant fluorochrome agent 4′,6-diamidino-2-phenylindole (DAPI) to detect DNA remnants. **(C)** Immunodetection of the Schwann cell marker S-100. **(D)** Immunohistochemical evaluation of the cytoskeletal protein neurofilament (NFL). **(E)** Immunodetection of the vimentin (VIM) as cytoskeletal fibroblast protein.

Concerning the histological characterization of the generated acellular grafts, we first evaluated their general histological structure with hematoxylin and eosin (HE) staining. This staining revealed the typical histological pattern of the nerve in NAT group (used as control) which was relatively well-preserved after the decellularization procedures used, but with certain differences (Figure 1A). Regarding the stromal organization, it was clearly identified in all DPNAs. Better organized and well-defined histological pattern were obtained within P1 and P2 groups. Surprisingly, histological analysis revealed some structural disruption and condensations in SD and specially P3 groups (Figure 1A). In relation to the nuclei preservation, HE and especially DAPI stainings did not reveal the presence of cell nuclei within the three novel protocols (P1, P2 and P3), but a weak positive reaction was observed in SD group (Figure 1B).

With the aim of determining whether some cellular cytoplasmic proteins are still remaining following decellularization procedures, immunohistochemistry for S-100 (SCs), NFL (axons) and vimentin (VIM, SCs and fibroblasts) were performed (Figures 1C–E **).** In general terms, all DPNAs showed a considerable decrease in the positive reaction for these proteins as compared to the NAT group. Immunoreaction for S-100 was less abundant in SD group than in the three new DPNAs. Regarding the NFL, this cytoskeletal protein was found in all DPNAs, and it was more efficiently removed in SD and P1 groups than in P2 and P3 groups. When VIM was assessed, no evident remnants of this cytoskeletal protein was found within all experimental conditions (Figure 1E).

**FIGURE 2:**
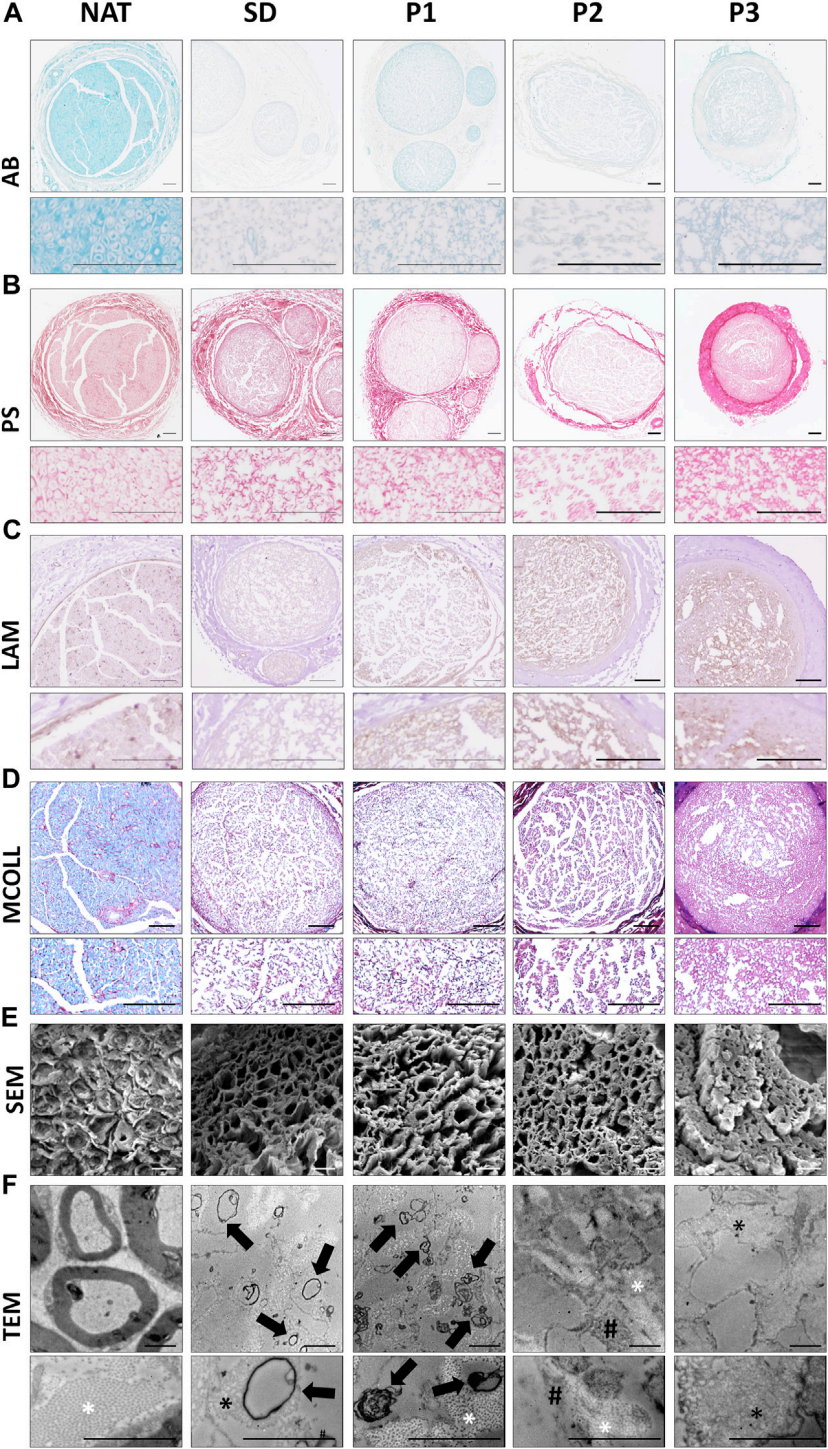
Histological, immunohistochemical and ultrastructural evaluation of the ECM and myelin content in the DPNAs. **(A)** Acid proteoglycans evaluation by Alcian blue (AB) histochemical method. **(B)** Histochemical identification of fibrillar collagens using Picrosirius staining (PS). **(C)** Immunohistochemistry for the basal membrane glycoprotein laminin (LAM); brown positive reaction contrasted by hematoxylin. **(D)** Simultaneous myelin and collagen network assessment by the myelin-collagen histochemical method (MCOLL); myelin (light-blue histochemical reaction) and collagen fibers (red positive reaction). **(E)** SEM and **(F)** TEM images of cross-sectional section of the DPNAs generated. Black arrows: Rest of myelin and cell membrane residues; * (white): well-organized collagen fibers; * (black): Disorganized collagen fibers; White arrows: Cytoplasmatic residues. Scale bar: **(A–D)** = 100 µm; **(E)** = 10 µm and **(F)** = 2 µm.

To specifically determine the impact of the decellularization procedures in the ECM, histochemical AB, PS and MCOLL, immunohistochemical (LAM) and ultrastructural (SEM and TEM) analyses were conducted (Figure 2).

Proteoglycans (PG) are important and especially abundant ECM molecules of nerve endoneurial compartment, but they must be removed after decellularization because of their negative impact on nerve regeneration (Wood and Mackinnon, 2015). AB PG staining showed a considerable decrease of these molecules in all DPNAs as compared with native nerves (Figure 2A). However, certain positive histochemical reaction remained in P1 groups. PS staining confirmed a well preservation of collagen network in all DPNAs (Figure 2B). The distribution pattern of PS histochemical reaction was adequate and comparable to NAT group in P1 group followed by SD group. In P2, collagen was also preserved and well distributed, but this histochemical reaction was weaker than the obtained with the other decellularized protocols. In the case of P3, PS reaction revealed a high condensation of the collagen content at the nerve surface without a clear definition between the epineurium and perineurium of obtained DPNAs (Figure 2B).

LAM is a basal membrane glycoprotein essential to stimulate Schwann cell function and migration during nerve regeneration. LAM preservation was studied by immunohistochemistry revealing an important preservation following decellularization in all DPNAs. These results were especially positive within the new protocols (P1, P2 and P3 groups) in which relatively well-defined endoneurial tubes were found (Figure 2C).

Regarding the myelin content, it is important to achieve an adequate removal of this component as myelin could delay nerve tissue regeneration process. As myelin is important, it was evaluated by histochemical and ultrastructural analyses. MCOLL staining confirmed an important removal of the myelin content in all DPNAs as compared to NAT control (Figure 2D) and we observed only some staining in SD and P1 groups. Moreover, MCOLL staining confirmed the collagen preservation observed with PS technique as expected.

Ultrastructural analyses by SEM showed the 3D pattern and porosity of the acellular matrix obtained. Adequate, consistent and well-defined endoneurial tubes were clearly obtained with P2 and P1 protocols with P2 and P1 protocols, being these results even better than those observed in SD group (Figure 2E). In line with light microscopy results, the 3D matrix obtained with protocol P3 was not optimal showing condensation of the ECM and collapse of the endoneural compartment resulting in a low degree of porosity. Concerning the myelin content, SEM did not reveal clear signs of myelin remnants (Figure 2E). When DPNAs were analyzed by TEM, this method confirmed the impact of the decellularization protocols on cellular, myelin and ECM component integrity. TEM was sensible enough to reveal the presence of some myelin or cell membrane residues in SD and P1 groups but not in P2 and P3 (Figure 2F). Referring to the preservation of the ECM, in P1 and P2 groups TEM confirmed a good preservation and organization of the collagen network as well as the negative impact of the decellularization process in SD and P3 groups (Figure 2F).

#### 3.1.2 DNA and glycosaminoglycans biochemical profile of DPNAs

**FIGURE 3:**
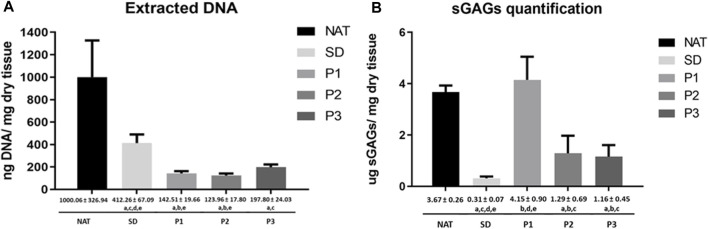
Quantitative biochemical analyses of DNA and sGAGs in DPNAs. **(A)** Quantification of extracted DNA. **(B)** Quantification of total sGAGs extracted. Graphical and numeric representation were expressed as mean values ± standard deviation values (error bars). Statistically significant differences (*p* < 0.05) were determined with the Mann–Whitney test and indicated as follows: a = vs. NAT group. b = vs. SD group. c = vs. P1. d = vs. P2 and e = vs. P3.

Biochemical kit assays were used to measure the total amount of DNA and sGAGs in the DPNAs generated and controls. The amount of DNA and sGAG observed in each experimental group is summarized in Figures 3A, B. Regarding the DNA content, it was significantly reduced following decellularization as compared to NAT group. In addition, the new decellularization protocols (P1, P2 and P3) showed significantly lower values than SD group (*p* < 0.05). Interestingly, DNA values were especially lower with P2 and P1 procedures, without significant differences between them (*p* = 0.175), but the differences between P1 and P2 vs. P3 were statistically significant (*p* < 0.05). Besides that, total sGAG was significantly reduced in SD, P2 and P3 groups (*p* < 0.05) as compared to NAT and P1 group. The method did not reveal a reduction of sGAGs in P1 being these findings in line with AB staining results. Surprisingly, SD method showed the lowest amount of sGAG values in this study being the differences with P2 and P3 statistically significant (*p* < 0.05). Finally, a comparable amount of sGAGs was obtained in P2 and P3 groups (*p* > 0.05).

#### 3.1.3 Tensile properties of DPNAs

**FIGURE 4:**
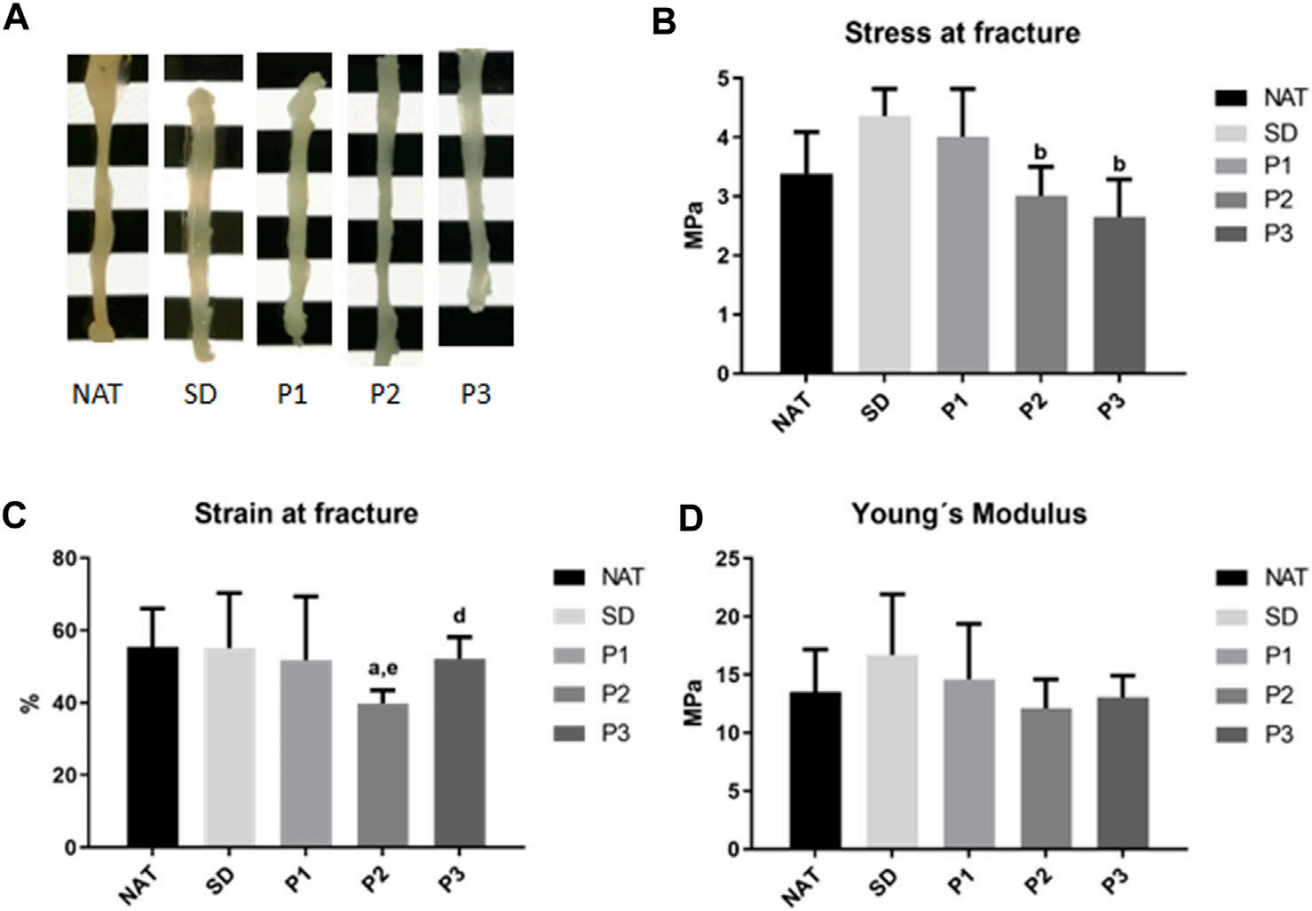
Macroscopic aspect and tensile properties of the DPNAs generated. **(A)** Macroscopic images of the different study groups. **(B)** Stress at fracture, **(C)** strain at fracture and **(D)** Young’s modulus biomechanical results were obtained after the tensile test of the DPNAs and native nerves. Statistically significant differences (*p* < 0.05) were determined with the Mann–Whitney test and indicated as follows: a = vs. NAT group. b = vs. SD group. c = vs. P1. d = vs. P2 and e = vs. P3.

To determine the impact of the decellularization on the biomechanical properties of the DPNAs, macroscopic appearance and tensile tests were conducted (Figure 4; Supplementary Table S2). The macroscopic analysis of the DPNAs revealed, in function of the protocol used, some differences in degree of transparency (Figure 4A), being SD the most transparent followed by P3. The tensile test showed that none of the obtained DPNAs presented significant differences (*p* > 0.05) to NAT group in stress at fracture values (Figure 4B). The only difference was found with P2 (3.01 ± 0.49 MPa) and P3 (2.65 ± 0.64 MPa) presenting significantly lower stress at fracture values than the SD group (4.36 ± 0.46 MPa, *p* < 0.05), which lightly (but not significantly) increased its rigidity respect to the NAT group (3.39 ± 0.70 MPa, *p* > 0.05). Remarkably, the values of strain at fracture were similar in all groups except P2 (39.76% ± 3.68%), which showed a significant decrease (*p* < 0.05) when it was compared with NAT (55.38% ± 10.64%) and P3 (52.16% ± 5.92%) groups (Figure 4C). In case of the Young’s modulus no significant differences were found among the tested groups (*p* > 0.05) (Figure 4D).

#### 3.1.4 *Ex-Vivo* cytocompatibility

In order to evaluate the *ex-vivo* biocompatibility of the DPNAs generated, rat ADMSCs were seeded within the nerve endoneurial compartment. The cytocompatibility was determined at 48 h with L/D and WST-1 tests.

**FIGURE 5:**
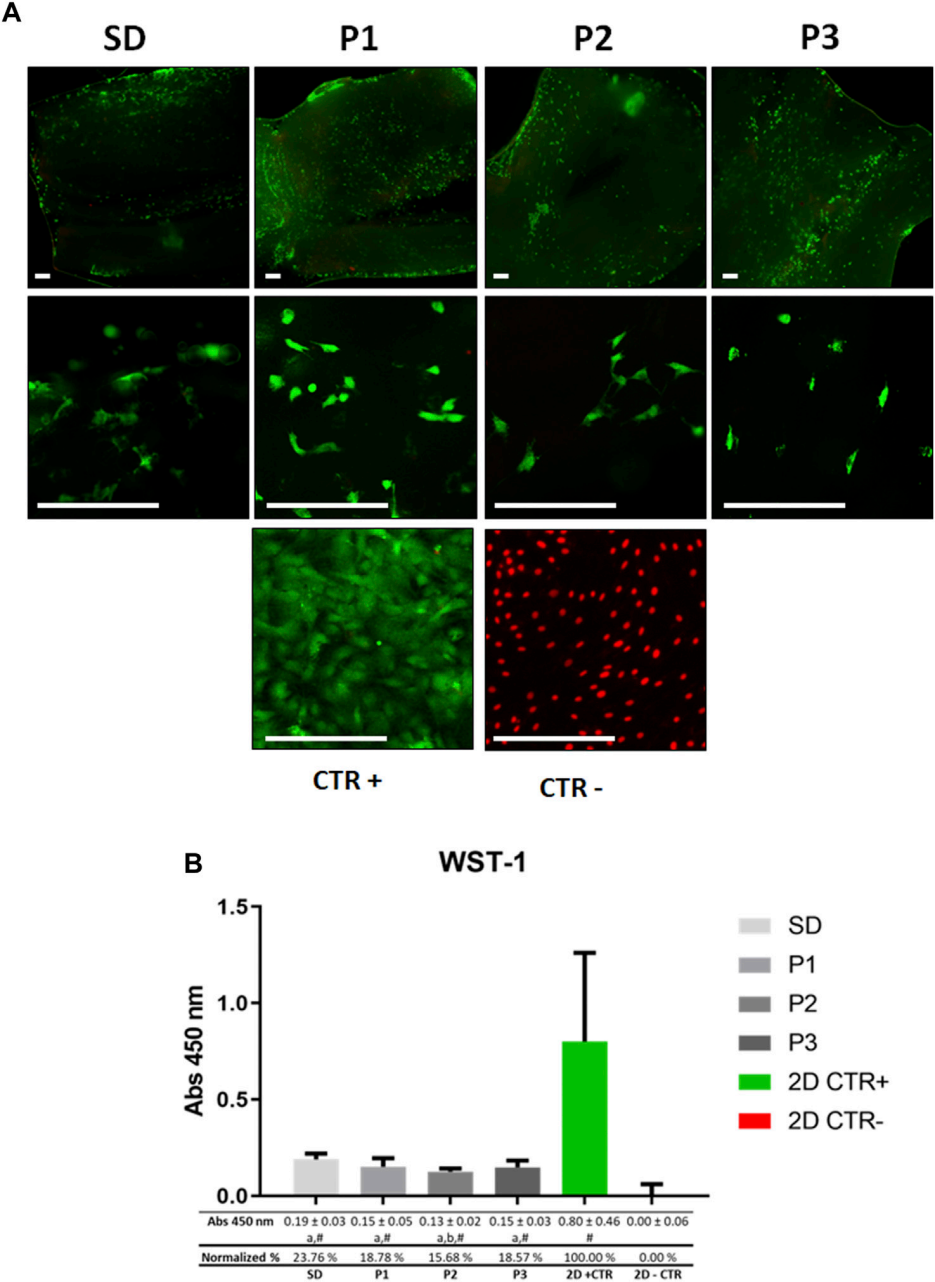
*Ex vivo* cytocompatibility assessment of DPNAs. The behavior of ADMSC cultured in the endoneurial part of DPNAs is shown for **(A)** Live&Dead (L/D) analysis and **(B)** WST-1cell metabolic assay. For **(A)** L/D assay non-cell seeded DPNAs of each decellularization method used were included as technical controls in order to confirm that these analyses were conducted with cell-free biomaterials and no cells remained after the decellularization process. Scale bar = 200 µm. For **(B)** mean values with their respective error bars corresponding to standard deviations were graphed. Statistically significant differences (*p* < 0.05) were determined with Mann–Whitney test as follows: # = vs. 2D – CTR; a = vs. 2D + CTR; b = vs. SD group; c = vs. P1 group; d = vs. P2 group; e = vs. P3 group.

The morphofunctional L/D assay confirmed the presence of a similar amount of viable rAMSC attached to the inner surface of all DPNAs (green fluorescence) evaluated (Figure 5A). However, SD group showed a lower number of attached cells. Regarding the presence of dead cells (red fluorescence), just few cells were identified within the DPNAs, while they were evident in the 2D negative control group. Dead cells cannot keep their cell-ECM interactions and thus most of them were removed during the staining procedure. Regarding the cell morphology, elongated cells were clearly observed in P1 and P2 groups, being these results similar to 2D positive control group. However, in SD and P3 groups cells presented shorter cytoplasmic extensions (Figure 5A).

The WST-1 assay (Figure 5B) showed a high level of cellular metabolic activity in all DPNAs generated, although these values were significantly lower than those obtained in the 2D positive CTR (*p* < 0.05) group. In general, normalized values oscillated between a 23.76% in SD group and 15,68% in P2 group (Figure 5B). Furthermore, when WST-1 values were compared among the different protocols used, only significant differences were found between SD and P2 groups (*p* < 0.05).

### 3.2 *In vivo* therapeutic efficacy of the DPNAs

Based on the results obtained from the *ex vivo* phase of this study, the P1, P2 and SD (as decellularized control) DPNAs were chosen for *in vivo* preclinical evaluation. In addition, nerve autograft (AUTO) technique was included as gold standard regeneration control group.

#### 3.2.1 Clinical and functional profile of laboratory animals

The functional evaluations at 15 weeks are summarized in Table 1. In reference to the degree of sensory recovery, all animals treated with DPNAs presented signs of sensory function recovery with Pinch test. Interestingly, only SD group resulted in significantly lower values (2.00 ± 0.89, *p* < 0.05) with respect to the AUTO group (3.00 ± 0.00), while differences among other operated animals were not significant (*p* > 0.05). The motor functional recovery profile, determined by the toe spread and SFI tests, confirmed the impairment of motor function as expected, being far to be comparable to the CTR group (*p* < 0.05). In this context, toe spread mean values were more favorable in AUTO group (1.33 ± 0.52) followed by P2 (1.17 ± 0.75) and P1 (1.00 ± 0.63) groups, being these results less favorable in SD group (0.83 ± 0.75). Additionally, similar results were observed with SFI calculation among operated animals, although P2 group showed the most favorable values (−71.02 ± 10.32) and P1 the lower ones (−84.00 ± 7.48). Please note that the autotomy excluded some animals from the SFI assessment (see details in the experimental section).

**TABLE 1:** Functional assessment after 15 weeks of sciatic nerve repair by using DPNAs and nerve autograft techniques. Statistically significant differences (*p* < 0.05) were determined with Mann–Whitney test as follows: a = vs. CTR; b = vs. AUTO; c = vs. SD group; d = vs. P1 group; e = vs. P2 group.

Group	Pinch test	Toe spread	SFI
CTR	3.00 ± 0.00	3.00 ± 0.00	−13.11 ± 8.46
AUTO	3.00 ± 0.00	1.33 ± 0.52^a^	−80.30 ± 10.30^a^
Sondell	2.00 ± 0.89^b^	0.83 ± 0.75^a^	−75.62 ± 3.36^a^
P1	2.17 ± 0.98	1.00 ± 0.63^a^	−84.00 ± 7.48^a^
P2	2.50 ± 1.22	1.17 ± 0.75^a^	−71.02 ± 10.32^a^

#### 3.2.2 Muscle morphometry

**FIGURE 6:**
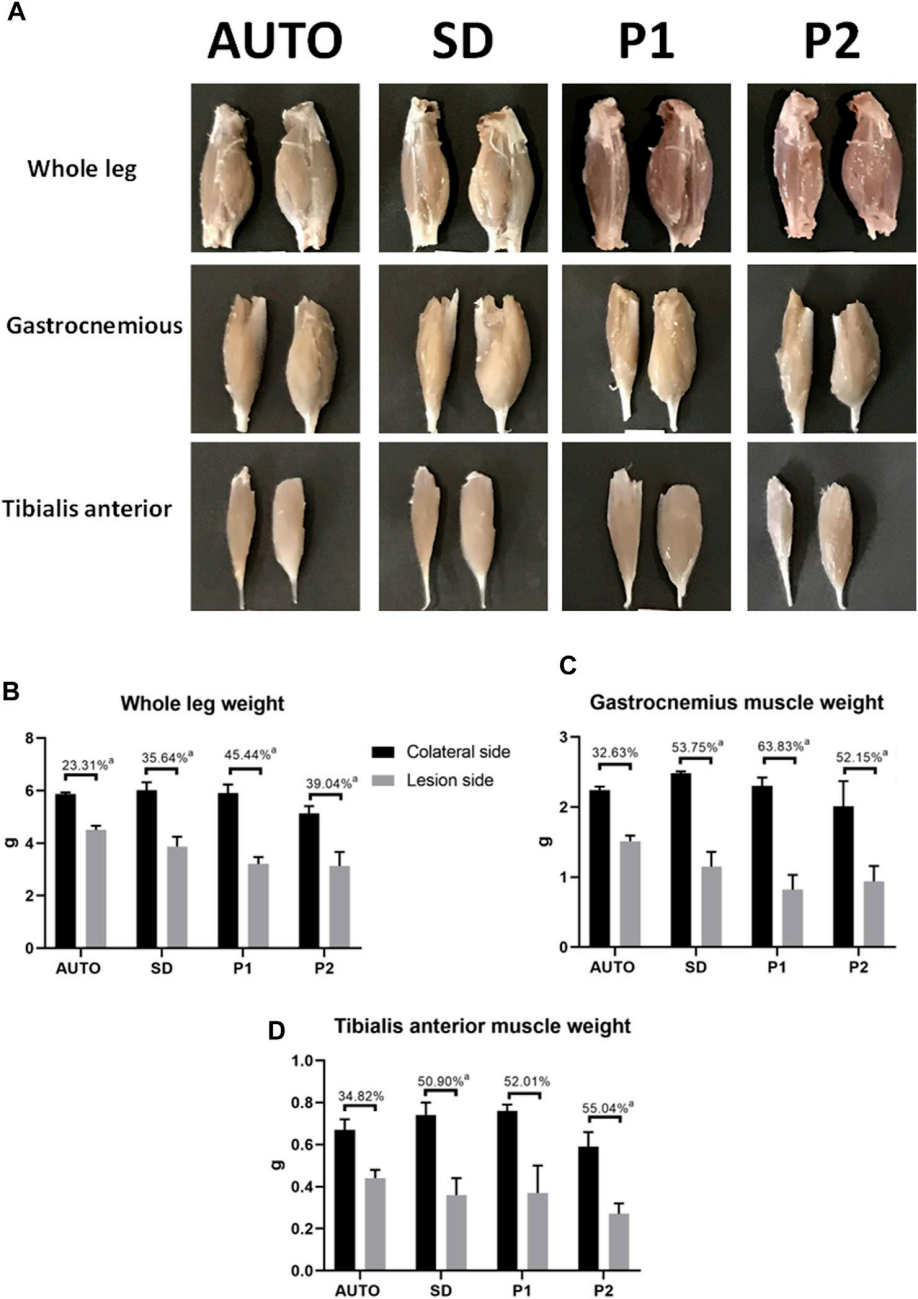
Macroscopic and graphical representation of muscular changes after 15 weeks of sciatic nerve repair by using DPNAs and autograft techniques. **(A)** Macroscopic analysis of the whole leg and the gastrocnemius and tibialis anterior muscles on the operated side (left) as compared to the contralateral legs (right) in all operated animals. Morphometric analyses comparing the lesioned side (gray) with the contralateral leg (black) of the whole leg **(B)**, the gastrocnemius **(C)** and tibialis anterior muscles **(D)** in all operated animals (AUTO, SD, P1, P2). The lost percentage is summarized in the upper part of each column group. Statistically significant differences (*p* < 0.05) were determined with Mann–Whitney test as follows: a = vs. CTR.

Macroscopic and morphometric analyses of the whole hindlimb and the *gastrocnemius* and *tibialis anterior muscles* showed a remarkable weight loss in the operated side compared to the contralateral side in all the operated animals (Figure 6; Supplementary Table S3). This general muscle atrophy (weight loss) was more evident and significantly higher in the whole hindlimb measurements of animals treated with DPNAs as compared to the AUTO group (*p* < 0.05). However, no significant differences were found among the DPNAs evaluated (*p* > 0.05). This fact was repeated when the *gastrocnemius* was evaluated, and the P2 group presented the lowest value among the DPNAs studied (52.15 ± 13.13 g) but observed differences among groups were not statistically significant (*p* > 0.05). Interestingly, when the *tibialis anterior* muscle weight loss was evaluated, favorable values were observed in AUTO group (34.82 ± 10.18 g), being the differences with SD and P2 groups statistically significant (*p* < 0.05). Moreover, *tibialis anterior* weight loss slight differences between DPNAs were not significant (*p* > 0.05) as well as between AUTO and P1 groups (Figure 6; Supplementary Table S3).

#### 3.2.3 Hematological and serological analyses

The quantitative hematological analyses revealed some differences in the hematological profile after 15 weeks of surgery (Table 2). The values of the parameters related to red blood cells (RBC, HGB, and MCV) and leucocytes (WBC, LYM, MXD, and NEUT) were closely comparable between the operated animals (AUTO, SD, P1 and P2) and the healthy animals (CTR group). However, some statistical differences were observed among the studied groups. Concerning the MCV count values, AUTO and SD groups showed a significant decrease respect to CTR group profile (*p* < 0.05). In addition, AUTO and P2 groups had significantly lower values for MXD count and % NEUT than CTR group (*p* < 0.05). Interestingly, in the case of the MPV the CTR group presented values (10.13 ± 0.84) outside the standard physiological range for female rats described by Kampfmann et al. (Kampfmann et al., 2012) (6.7–7.8). In addition, these values were significantly higher values (*p* < 0.05) than most groups except P1. In fact, P1 showed higher values (*p* < 0.05) of MPV than AUTO and P2 groups (Table 2).

**TABLE 2:** Hematological and biochemical profile after 15 weeks of peripheral nerve repair.

Application	Parameters	Units	CTR	AUTO	SD	P1	P2	Significant differences (*p* < 0.05)
Erythrocytes	RBC	10^6 mL^-1^	7.60 ± 0.48*	7.68 ± 0.62	7.43 ± 0.39*	7.93 ± 0.67*	7.89 ± 0.33*	−
	HGB	g/dL	14.27 ± 0.74	14.33 ± 0.71	13.40 ± 0.67	14.15 ± 1.38	14.83 ± 0.8	SD vs. P2
	MCV	fL	57.10 ± 0.69*	55.17 ± 0.95	55.07 ± 1.00	55.95 ± 2.20	56.52 ± 2.42	CTR vs. AUTO, SD
Leucocytes	WBC	10^3 mL^-1^	6.60 ± 1.47	9.85 ± 3.05*	7.35 ± 4.60	7.95 ± 3.82	7.77 ± 1.05	−
	LYM	%	90.73 ± 4.79	82.00 ± 8.47	81.83 ± 10.49	83.70 ± 5.42	82.62 ± 6.01	−
		10^3 mL^-1^	6.07 ± 1.59	7.97 ± 2.38	5.68 ± 2.50	6.62 ± 3.1	6.40 ± 1.04	−
	MXD	%	14.70 ± 5.72	15.65 ± 8.75	16.00 ± 7.58	13.30 ± 5.72	15.62 ± 5.36	−
		10^2 mL^-1^	2.00 ± 3.46	16.5 ± 10.75	12.5 ± 16.9	11.67 ± 9.33	12.33 ± 4.76	CTR vs. AUTO, P2
	NEUT	%	5.23 ± 1.72	2.35 ± 0.86	4.83 ± 3.09	3.00 ± 3.54	1.77 ± 0.89	CTR vs. AUTO, P2
								SD vs. P2
		10^2 mL^-1^	3.33 ± 2.08	2.33 ± 1.51*	2.00 ± 1.55*	1.67 ± 1.21*	1.33 ± 0.52*	−
Platelets	MPV	fL	10.13 ± 0.84*	6.28 ± 0.21*	7.68 ± 1.38	7.87 ± 1.34*	6.35 ± 0.20*	CTR vs. AUTO, SD, P2
								P1 vs. AUTO, P2
Liver	ALTL	10^2 U/L	1.16 ± 0.72	0.49 ± 0.12	1.41 ± 1.36	0.49 ± 0.17	0.41 ± 0.09	SD vs. P2
	ASTL	10^2 U/L	2.72 ± 1.77	1.23 ± 0.28	3.09 ± 1.95	1.36 ± 0.2	1.15 ± 0.19	SD vs. AUTO, P2
	BILT3	mg/dL	1.91 ± 0.62	2.81 ± 0.82	1.84 ± 0.59	1.95 ± 1.14	1.77 ± 0.84	−
Kidney	CRE	mg/dl	0.55 ± 0.11	0.49 ± 0.08	0.55 ± 0.11	0.45 ± 0.06	0.44 ± 0.07	−
	UREAL	mg/dl	31.33 ± 4.12	32.22 ± 4.62	33.33 ± 3.28	32.18 ± 6.28	32.07 ± 7.73	−
Lipid profile	LDLC	mg/dl	9.52 ± 1.31	8.66 ± 5.3	9.20 ± 4.33	7.93 ± 3.14	10.21 ± 2.54	−
	HDLC	mg/dl	41.19 ± 22.61	58.91 ± 20.48	50.09 ± 11.13	58.91 ± 10.63	67.6 ± 8.52	SD vs. P2
	TRIGL	10^2 mg/dL	1.97 ± 0.83	1.77 ± 0.52	1.19 ± 0.25	1.34 ± 0.33	1.57 ± 0.64	AUTO vs. SD
Pancreas	AMYL	10^2 U/L	16.67 ± 1.59	19.13 ± 1.96	15.09 ± 2.2	16.02 ± 2.12	17.2 ± 1.02	AUTO vs. SD, P1

*Note: Values for each parameter are shown as mean values ±standard deviation, and *p* < 0.05 were considered statistically significant. Asterisk (*) indicates values that differed from the normal range for female Wistar rats according to the available literature. (Kampfmann et al., 2012).

The serological biochemical values showed no statistically significant differences between the operated animals and the CTR group neither out of range values (liver, kidney, pancreas, and lipid profile-related biomarkers) suggesting no toxicity or adverse effects due to the grafts used. Liver enzymes (ALTL and ASTL) were slightly increased in SD group while all other operated groups showed a slight but not significant decrease when were compared with CTR group (*p* > 0.05). Significant differences were found in ALTL values between SD vs. P2 (*p* = 0.037) and in ASTL values between SD vs. P2 and AUTO (*p* < 0.05). Additionally, BILT and kidney-related serological markers, such as CRE and UREA, showed no significant difference (*p* > 0.05) between the studied groups. On the other hand, the lipid profile of operated animals did not differ as compared to CTR group (*p* > 0.05). However, the SD group showed significantly lower HDLC and TRYGL values as compared to P2 and AUTO groups respectively (*p* < 0.05). Regarding the pancreas-related enzyme AMYL, the AUTO group presented significantly higher values than SD and P1 group (*p* < 0.05), but not to the CTR group (*p* > 0.05). Finally, mean ± standard deviation values of each hematological or biochemical parameter and the statistically significant differences for all comparisons are indicated in the Table 2.

#### 3.2.4 Histological assessment of nerve tissue regeneration

**FIGURE 7:**
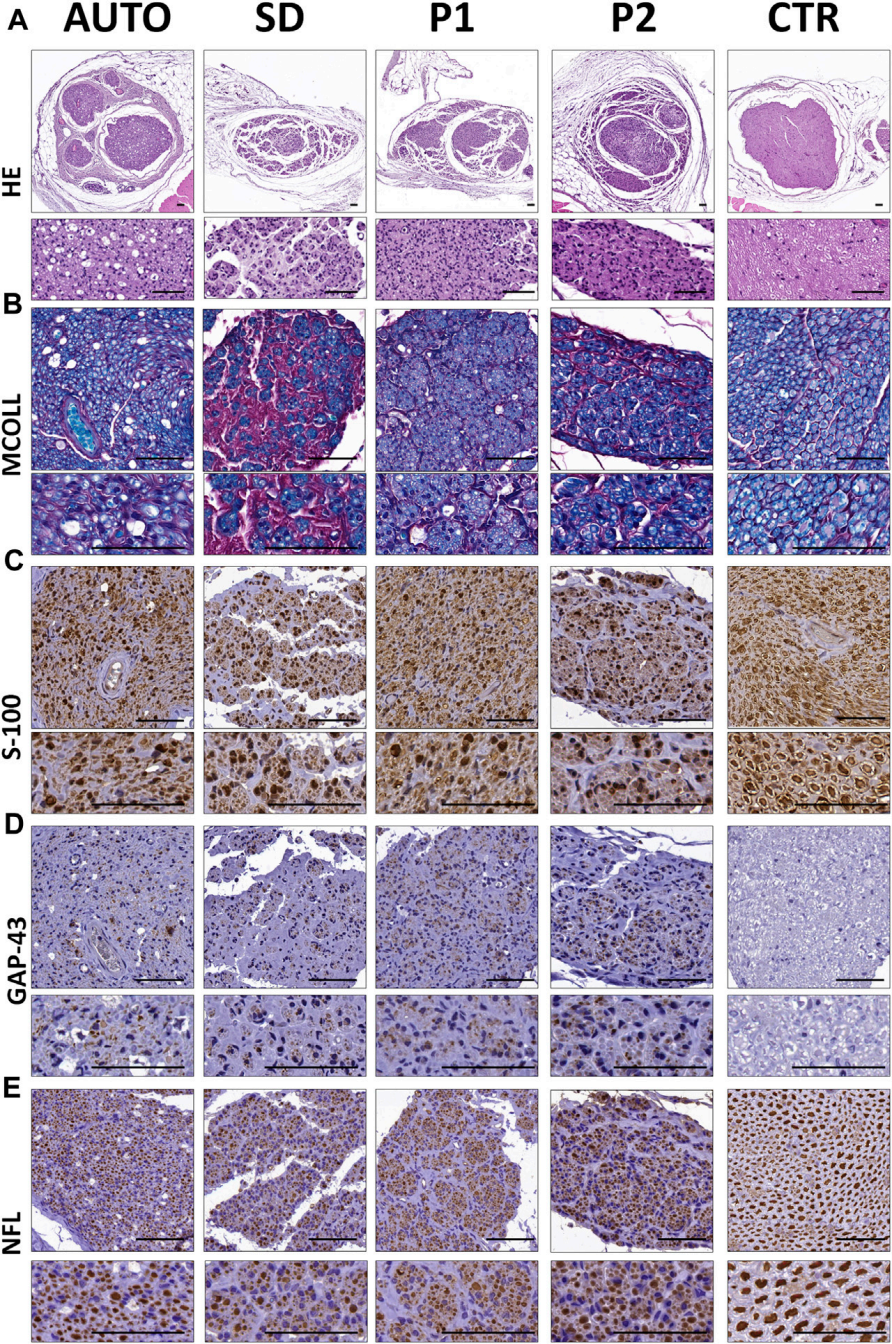
Peripheral nerve regeneration histological profile of the central region of the implanted grafts after 15 weeks of surgery. **(A)** General morphology of the operated and control nerves assessed by hematoxylin-eosin (HE) histochemical method. **(B)** Evaluation of myelin and collagen fibers content and distribution was performed by myelin-collagen (MCOLL) histochemical method. **(C)** Schwann cell identification by S-100 marker immunostaining. **(D)** Immunodetection of GAP-43 marker to assess the newly nerve fibers generated. **(E)** Mature nerve fibers generated were showed by neurofilament (NFL) immunohistochemical staining. Scale bar = 50 nm.

Histological analyses of the central part of the implanted grafts confirmed the presence of an active nerve regeneration process in all implanted grafts (Figure 7). General histological assessment conducted with HE (Figure 7A) revealed that the nerve regeneration process mainly occurs within the intrafascicular compartments of implanted grafts. In AUTO group the regenerated tissue was homogeneously distributed differing from the microfascicular pattern observed with the use of DPNAs. Regenerating microfascicles were more dispersed, thinner and surrounded by more abundant ECM in SD group than in P1 and P2 groups which were more comparable to AUTO group (Figure 7A).

The simultaneous evaluation of the content and distribution of myelin and collagen network was carried out by MCOLL staining (Figure 7B). This method clearly showed the myelination of the regenerating PNFs in all operated animals. The myelin content was clearly more abundant in the AUTO followed by P1 and P2 and finally the SD groups. Regarding the pattern of the regenerating tissue, MCOLL confirmed the microfascicular pattern observed by HE and revealed some differences in the collagen content among groups. In SD group, a high amount of collagen fibers was observed surrounding the thin and disperse regenerating microfascicles (Figure 7B) indicating certain degree of fibrosis. In contrast, a well-defined and organized collagen network was observed in AUTO, P1 and P2 (Figure 7B).

S-100 protein immunohistochemistry confirmed an abundant amount of Schwann cells within the regenerating nerve tissue in all operated animals (Figure 7C). These cells were consistent and homogeneously distributed in the AUTO and P1 groups. In the case of SD and P2 DPNAs, these cells showed the fascicular pattern described above. When regenerating axons were evaluated, GAP-43 protein confirmed the presence of immature axons in all groups, being slightly more abundant in the P2 group (Figure 7D). NFL immunohistochemistry confirmed a clear and active axonal regeneration in all operated animals. Regenerating axons were abundant and homogeneously distributed in the AUTO group followed by P1, P2 and lastly SD groups (Figure 7E). Regarding the histological pattern, NFLs staining confirmed the microfascicular organization within the DPNAs, being these results in line with HE, MCOLL and S-100 stainings (Figure 7).

#### 3.2.5 Quantitative and ultrastructural assessment of nerve regeneration

**FIGURE 8:**
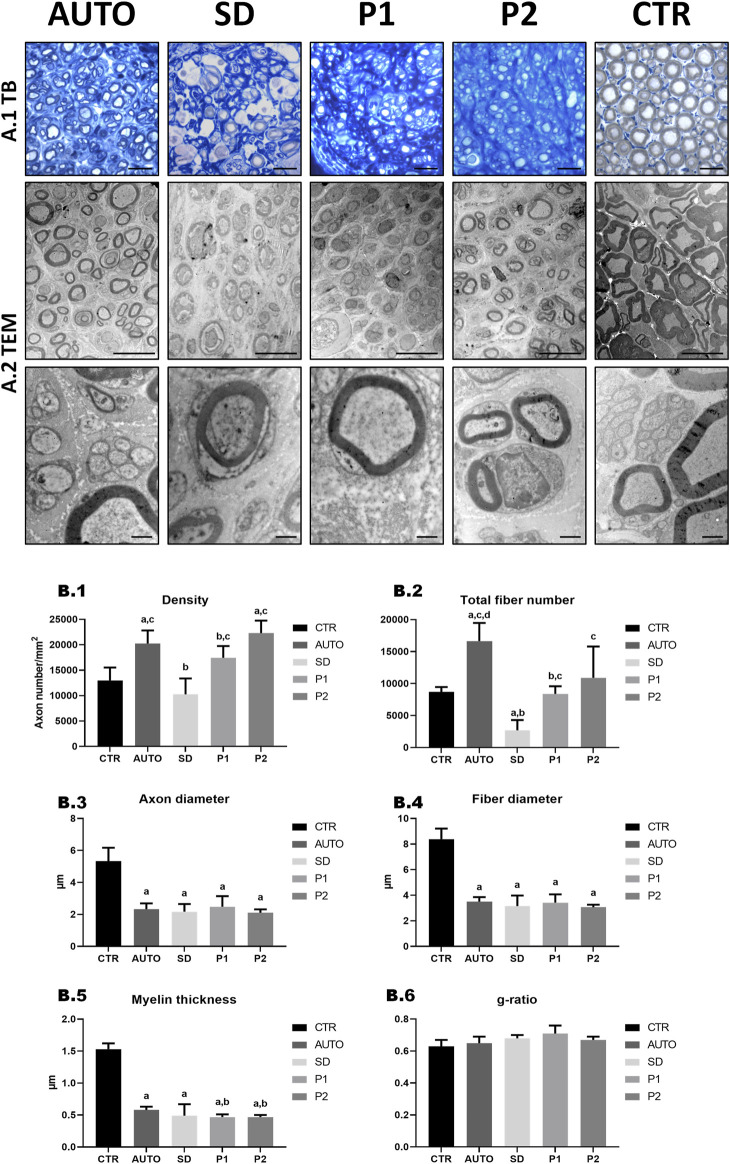
Quantitative and ultrastructural assessment of nerve regeneration in the distal region of the graft. **(A-1)** General morphology evaluation by toluidine blue (TB) staining. **(A-2)** Representative TEM images of cross-sectional section. Scale bar: **(A-1)** = 20 µm; **(A-2)** = 10 nm low magnification and 1 nm high magnification images. **(B)** Quantitative histomorphometry of the DPNAs were performed. **(B-1)** Density of fibers; **(B-2)** Total fiber number; **(B-3)** Axon diameter; **(B-4)** Fiber diameter; **(B-5)** Myelin thickness; **(B-6)** G-ratio (axon diameter/fiber diameter). Statistically significant differences (*p* < 0.05) were determined with Mann–Whitney test as follows: a = vs. CTR; b = vs. AUTO; c = vs. SD group; d = vs. P1 group; e = vs. P2 group.

Histomorphometrical and ultrastructural analysis of the distal part of operated sciatic nerves were performed after a healing period of 15 weeks (Figure 8) and the stereological parameters are summarized in Supplementary Table S4. Histological images of transverse sections stained with toluidine blue (Figure 8A-1) showed the presence of regenerated nerve myelinated fibers in all the operated groups. Among the operated animals, SD group qualitatively showed less myelinated fibers than all other groups and still clear sign of degeneration. Moreover, TEM analysis (Figure 8A-2) better showed myelinated and unmyelinated fibers morphology and well-organized endoneurial collagen network in all groups.

For the quantitative histological assessment of nerve regeneration (Figure 8B; Supplementary Table S4), the different operated groups and CTR were evaluated comparing the density and the number of myelinated fibers and different size parameters (axon and fiber diameter, myelin thickness and g-ratio). As regards density, AUTO (20.26 ± 2.57 fibers 10^3^/mm^2^) and P2 (22.31 ± 2.45 fibers ·10^3^/mm^2^) presented significantly higher density of fibers (*p* < 0.05) than CTR (12.99 ± 2.53 fibers/mm^2^) in contrast to SD (10.27 ± 3.12 fibers ·10^3^/mm^2^) and P1 (17.45 ± 2.30 fibers ·10^3^/mm^2^), where did not differ significantly (*p* > 0.05). Evaluating the number of myelinated fibers, the AUTO group showed the highest total number of fiber (16.64 ± 2.83 fibers ·10^3^) and was the only group that was significantly superior (*p* < 0.05) to the CTR group. In addition, SD presented significantly lower values in density and total fiber number (*p* < 0.05) than the other operated groups. As regards size parameters, significantly lower values (*p* < 0.05) were found in axon and fiber diameter and myelin thickness in all operated animal groups compared to CTR. Moreover, AUTO (0.58 ± 0.05 µm) presented significant higher myelin thickness than P1 (0.47 ± 0.04 µm) and P2 (0.47 ± 0.03 µm) but curiously did not with SD group (0.49 ± 0.18 µm). Finally, no significant differences (*p* > 0.05) were found in the g-ratio between the different experimental conditions.

## 4 Discussion

Nowadays, the successful nerve tissue regeneration and subsequent functional recovery following long peripheral nerve injury repair is still a challenge (Carriel et al., 2014). Consequently, extensive efforts have been made to generate bio-artificial conduits to be used as effective alternative to the current gold standard technique, the nerve autograft. Despite of all the improvements in the production of bioengineered nerve substitutes, the structural and molecular complexity of these organs is not properly achieved yet.

In this context, DPNAs have emerged as a non-immunogenic, highly biomimetic and biocompatible tissue-specific substitutes for this purpose, supported by *ex vivo* and even *in vivo* promising studies (Lovati et al., 2018; Choudhury et al., 2020). Nevertheless, the scientific evidence demonstrates that it is still necessary to develop more efficient decellularization methods (Peters et al., 2021). Besides, new methods are focused on the improvement of two key aspects: an efficient cell removal (cell components and myelin) and an adequate preservation of the 3D structure, composition, and biomechanics of the resultant ECM. In this line, three novel decellularization methods were designed, fully characterized *ex vivo* and tested in the repair of 10 mm nerve defects in rats. For this purpose, different concentrations of non-ionic (Triton X-100), and ionic detergents (SDS or SDC) were rationally applied to promote the cellular lysis and myelin removal, while enzymatic treatment (RNase and DNase) or 3% PAA were included to specifically remove the nucleic acid content (Lovati et al., 2016; El Soury et al., 2021), as they are responsible of the immunological response *in vivo*.

Concerning the *ex vivo* characterization, the three novel methods were compared to the properties of the well-described SD method (Lovati et al., 2018). In this regard, the effectivity of the different decellularization methods was confirmed by using histological, ultrastructural, DNA quantification *ex vivo* assays.

The decellularization efficacy, assessed by histological and biochemical tests, achieved with the novel (P1, P2 and P3) and control (SD) DPNAs was in general satisfactory. Here, we evaluated the presence of cell nuclei and DNA content as in previous studies (Crapo et al., 2011; Shin et al., 2019). Moreover, the presence of some cell-specific cytoplasmic proteins (S-100, NFL and VIM) and the myelin content were studied by immunohistochemistry according to recent studies (Philips et al., 2018a; Philips et al., 2018b; El Soury et al., 2021; Garcia-Garcia et al., 2021).

The removal of those cytoplasmatic proteins was satisfactory with the novel (P1, P2 and P3) and the classic SD methods, except in case of the cytoskeletal NFL protein which was preserved after all decellularization procedures due to its high resistance to degradability by detergents as previously described (Lodish et al., 2006). Another cellular component that must be removed from the obtained DPNAs is the myelin, which was majorly removed in all experimental groups observed in MCOLL method and confirmed by the ultrastructural SEM images. However, TEM images showed that the myelin was totally removed only in P2 and P3, showing some myelin debris in SD and P1 groups. This fact was one of the main aspects of improvement from the previous decellularization method of Roosens et al. (Philips et al., 2018a) which could have an inhibitory effect in axonal regeneration (Philips et al., 2018b). In addition, the attempt to replace the use of nucleases by other cheaper reagents as PAA in P3, which could provide a substantial advantage in the subsequent industrial scale-up production, did not reach satisfactory DNA removal values upon decellularization. Unfortunately, observing the DNA extraction results, PAA did not achieve the effectivity of the enzymes when was compared with the analogous method P2, but neither when was compared to the softer decellularization method P1. This significant decrease of DNA content, even using a less aggressive protocol with lower detergent concentration, highlighted the importance of the enzymatic steps in the decellularization procedures as was already stated in previous studies (Sridharan et al., 2015; Shin et al., 2019).

The main goal of tissue decellularization is to obtain cell-free tissue-specific 3D matrices composed by essential ECM molecules, thus the assessment of the structure and main ECM molecules within the DPNAs generated is highly important (Keane et al., 2015; Williams, 2015; Shin et al., 2019). The histological and biochemical evaluation of sGAGs confirmed their considerable decrease following nerve decellularization with P2, P3 and SD procedures, but this decrease was less evident in P1 group. This fact could be related to the lower concentration of SDC used in P1 group, being these results in agreement with previous works (Philips et al., 2018a). The proteoglycans removal, such as chondroitin sulfate, is considered beneficial to the axonal outgrowth and nerve regeneration (Muir, 2010), therefore, the DPNAs generated in this study adequately meet with this criterion. Regarding the collagen network, histochemistry confirmed its adequate preservation and organization with P1, P2 and SD decellularization protocols, but not in P3 group. Surprisingly, in P3 group, where 3% PAA was used, histology revealed condensation of the collagen fibers within the epi- and perineural layers losing the well-defined histological pattern and porosity, adequately achieved by P1, P2 and SD procedures. It is well-known that collagens can be irreversibly denatured by the use of acid solutions. PAA has been used in tissue decellularization such as tendon and nerve (Lovati et al., 2016; El Soury et al., 2021), but this agent is also frequently used in denaturing collagens to generate gelatin (Perez-Puyana et al., 2019).

In this sense, and based on these results, it is recommended to avoid the use of acidic treatment (or adapt its concentration and time) if a collagen network needs to be well-preserved. Another important ECM molecule that should be preserved to promote nerve regeneration, is laminin. This glycoprotein supports Schwann cell migration and proliferation, being an essential substrate for the Büngner band formation process (Chernousov et al., 2008; Jessen and Mirsky, 2016). Here, an abundant and properly distributed preservation of laminin was successfully achieved with the three new decellularization techniques, being these results considerably better than SD group and comparable to use of similar procedures (El Soury et al., 2021; Garcia-Garcia et al., 2021). Finally, the well-preservation of the ECM obtained with P1, P2 and SD, as well as the alterations in P3, were well-confirmed by ultrastructural analyses (SEM and TEM).

Thirdly, the removal of certain ECM components by the different decellularization procedures could be associated with changes in the physical and biological properties of the generated DPNAs (El Soury et al., 2021; Garcia-Garcia et al., 2021; Holland et al., 2021), something that was evaluated by the physical and biomechanical characterization of the DPNAs. These tests are essential tools to determine the suitability of the nerve substitutes to clinical practice and, interestingly, the decellularization methods described here did not significantly affect overall biomechanical properties of the nerves, obtaining biomechanical properties comparable to the native nerves used as control. These results are in line with other published studies that used similar decellularization protocols (El Soury et al., 2021; Garcia-Garcia et al., 2021). Concerning the biological properties of the generated DPNAs, they are composed by a natural and properly organized ECM and therefore differ from nerve substitutes composed by synthetic biomaterials which, according to the literature, are less biocompatible (Williams, 2008; Zor et al., 2019). However, it was observed that the use of chemical agents during decellularization might affect the biocompatibility of the generated matrices (Keane et al., 2015; Philips et al., 2018b). In fact, it was reported in other studies (Xu et al., 2014; Kawecki et al., 2018) that if remnants of some detergents, specially SDS and Triton X-100, are still in the scaffold after the decellularization process, they could commit cellular growth and graft colonization. In this work, the cell-biomaterial interaction analyses (cytocompatibility assays) confirmed that the different DPNAs produced supported the adhesion and growth of numerous viable and metabolically active ADMSCs under standard culture conditions. These positive results, even using SDS and Triton X-100, are supported by previous studies in which detergent rinse and clearance are vital steps to ensure cellular growth (Wakimura et al., 2015; Garcia-Garcia et al., 2021; Sanchez-Porras et al., 2021).

Finally, the histological, biomechanical and biological analyses demonstrated that the P1 and P2 generated DPNAs have optimal structural, biomechanical and biological properties to be tested in the repair of 10 mm nerve gaps in rats. The therapeutic efficacy of these products was comprehensively assessed by clinical-functional, hematological and serological, muscle morphometrical and comprehensive histological analyses.

Clinical and functional assessments are a keystone in the quality control of peripheral nerve regeneration (Vleggeert-Lankamp, 2007; Carriel et al., 2014). The rat sciatic nerve, as a repair-regeneration model, is associated to different clinical implications that are typically evaluated by clinical tests such as the Pinch test for sensitive evaluation and the toe spread test for motor recovery. Here and according to the literature (Bain et al., 1989; van Neerven et al., 2012; Chato-Astrain et al., 2018; Chato-Astrain et al., 2020b), the sensory recovery obtained by the pinch test, was also correlated with a better motor toe spread results. However, in case of motor recovery SFI test, this tendence is not so clear, showing partial recovery in all operated animals and getting the better results in P2 followed by the AUTO group, but not being comparable with the healthy control group as expected. These motor recovery SFI results of P2 were slightly superior to other decellularization methods such as our previous study using the Roosens method (Chato-Astrain et al., 2020b) and Liu et al. (2011). Non-etheless, some animals were excluded from the SFI test due to the presence of different degrees of autotomy. This fact is one of the main drawbacks related to the rat sciatic nerve injury model which may interfere in the interpretation of the functional results (Vleggeert-Lankamp, 2007; Geuna, 2015; Lovati et al., 2018). In fact, SFI as the only indicator of motor function recovery is not sufficient and other functional test must be done in parallel as the toe spread test that we included or ideally electromyographic studies (Vleggeert-Lankamp, 2007; Lovati et al., 2018). Ultimately, our results suggest that the presence of autotomy might be related to a lower sensory recovery and thus the nerve tissue regeneration, something that was later on confirmed by the histological and histomorphometrical analysis.

Another key aspect in tissue engineering is to produce a minimal host immune response to engineered grafts once implanted (Carriel et al., 2014; Williams, 2015). In this case, decellularized allograft could evoke different degrees of host immune response by an incomplete removal of cellular material, the presence of chemical residues, molecular modifications of the ECM molecules, or even due to the sterilization procedure used (Keane et al., 2015; Philips et al., 2018b). Therefore, in this study hematological analyses were performed and quantitatively analyzed to evaluate the host immune response to implanted grafts (Kampfmann et al., 2012). No changes were found in most of the erythrocytes-related parameters (RBC, HGB and MCV) among the operated animals. In case of the leucocytes profile (WBC, LYM, MXD, and NEUT), which are directly related to immune host response, no differences were found in LYM values. Nevertheless, it was observed an increase of MXD values in all operated animals as compared to CTR group, but differences were only significant in P2 and AUTO groups, curiously the same groups that also showed a significant decrease of NEUT values as compared to CTR group. These few differences could be related to an active remodeling process in operated animals, particularly in these two groups (AUTO and P2 groups). MXD results may have a logical explanation, since in the autograft group an important recruitment of macrophages, for the removal of myelin and cellular debris, must take place and a similar process could occur within the DPNAs, being more evident and significant in P2. Overall hematological, and specially WBC values, confirmed no signs of host immune response against implanted grafts, and therefore the differences would be more related to a remodeling of the graft ECM to promote tissue regeneration within it. Moreover, the biochemical profile of each group was assessed to discard any affections or disfunctions of vital organs following nerve repair. In fact, there were no significant differences between the CTR group and all the operated animals in liver, kidney, and pancreas associated parameters. Hematological and serological results suggest that the DPNAs used are highly biocompatible and safe to be used in nerve repair. Furthermore, overall results are in line with the fluctuation, stabilization and even histological correlation of certain WBC values following the subcutaneous implantation of diverse biomaterials (Campos et al., 2020; Campos et al., 2021) or nerve repair in rats (Chato-Astrain et al., 2020b).

Evaluation of muscle function recovery is one of the main assessments of the progress of nerve regeneration. The lack or incomplete innervation of the muscles causes neurogenic atrophy (Lovati et al., 2018). During early stages following nerve repair, a progressive decrease of muscle weight occurs (El Soury et al., 2023), but depending on the graft used signs of recovery and less atrophy can be obtained over time (Zhao et al., 2014). In our study, the significantly lower degree of atrophy was obtained in the AUTO group as expected, as compared to all the DPNAs groups. Furthermore, no significant differences were found among the DPNAs studied. These results agree with most articles in which better muscle trophism was obtained in animals treated with nerve autografts than bioengineered nerve substitutes (Debski et al., 2021; Yu et al., 2021) and DPNAs (Zheng et al., 2014; Chato-Astrain et al., 2020b; Qian et al., 2022).

Histology is considered one of the most useful and accurate quality control in peripheral nerve repair/regeneration studies (Carriel et al., 2014; Chato-Astrain et al., 2020a). In this study, histological analyses confirmed a consistent and highly active nerve tissue regeneration process in the central portion of implanted grafts after 15 weeks. In all studied groups, the MCOLL histochemical method revealed different degrees of myelination of the newly-formed nerve fibers, which clearly demonstrate the correct establishment of the Schwann cell-axonal functional unit (Geuna et al., 2009). These findings were corroborated by the abundant positive reaction for Schwann cells (S-100), and axonal remodeling and cytoskeletal proteins (GAP-43 and NFL). Regarding the histological pattern, the nerve regeneration process was more abundant and homogeneous in animals from AUTO and P2 groups whereas an evident microfasciculation (newly-formed nerve fascicles) and signs of fibrotic response characterized the nerve regeneration process in SD and to a lesser degree P1 group, being these findings in accordance with previous studies (Chato-Astrain et al., 2020b). Moreover, histomorphometrical analyses, another key quality controls in the field, provided objective and quantitative values of the nerve regeneration process following peripheral nerve repair (Raimondo et al., 2009; Chato-Astrain et al., 2020a). These analyses confirmed the microfasciculation phenomena typical of regenerating nerves with smaller myelinated fibers and thinner myelin sheaths than healthy nerves (Geuna et al., 2009; Raimondo et al., 2009; Ronchi et al., 2015; Lovati et al., 2018). These observations were accurately confirmed by TEM analysis. Moreover, the evaluation of density and number of myelinated fibers showed that AUTO and P2 groups presented higher density than CTR and, while the number of myelinated nerve fibers in AUTO group is higher than CTR, the SD group showed a significantly lower number of fibers than CTR. The new generated decellularization methods (P1 and P2) presented significant superior results in fiber density and total number to the classic SD group, but only P2 presented comparable histomorphometrical profile to AUTO group. However, as expected the newly-formed axons and fibers did not acquire the diameter they had prior to the injury (Raimondo et al., 2009; Ronchi et al., 2015). Finally, the differences in the amount and pattern of regenerating nerve tissue and histomorphometrical profile achieved through the use of the different DPNAs were correlated with the *ex vivo* properties of the generated grafts. Indeed, the nerve tissue regeneration process was more favorable, and comparable to AUTO group, when animals were treated with DPNAs generated by P2, which showed an efficient decellularization and presented an adequate preservation and organization of ECM with abundant collagen fibers and laminin, both highly essential ECM molecules for nerve tissue regeneration (Chernousov et al., 2008).

In conclusion, the complete *ex vivo* characterization of the novel DPNAs (P1, P2 and P3) confirmed an efficient decellularization and ECM preservation with P1 and P2 protocols. These new DPNAs were even better, in terms of decellularization, structure, biomechanical and biological properties than those generated by SD or P3 procedures. Furthermore, the *in vivo* evaluation of P1 and especially P2 DPNAs allowed to conduct an adequate nerve surgical repair keeping the continuity of the repaired nerves after 15 weeks. From the clinic-functional point of view, these novel DPNAs showed highly promising results which in the case of P2 DPNAs were closely comparable to the efficacy of nerve autograft used as control. Hematological and histological analyses confirmed that these new P2 DPNAs supported and guide a highly active nerve tissue regeneration process without any adverse effects. Furthermore, comparable histological profile was obtained by using P2 DPNAs and nerve autograft, being both superior to the efficacy observed by the use of SD and P1 DPNAs. Finally, overall *ex vivo* and *in vivo* comprehensive analyses support the potential usefulness of the novel P2 DPNAs in peripheral nerve repair. These new substitutes were comparable, but not superior, to the nerve autograft technique. However, it is still necessary to conduct more basic research to determine the efficacy of these products in the repair of large nerve defects. In addition, future studies will explore diverse functionalization strategies in order to improve the biological properties and regeneration capability of these matrices for the potential clinical use of these novel advanced therapy products.

## Data Availability

The original contributions presented in the study are included in the article/Supplementary Material, further inquiries can be directed to the corresponding authors.

## Glossary

**AB:**: Alcian Blue
**ADMSC:**: adipose-derived mesenchymal stem cells
**ALT:**: alanine transaminase
**AMYL:**: amylase
**AST:**: aspartate aminotransferase
**AUTO:**: autograft technique
**BILT:**: total bilirubin,
**CRE:**: creatinine
**CTR:**: healthy control group
**DAPI:**: 4′,6-diamidino-2-phenylindole
**DMEM:**: Dulbecco’s modified Eagle medium
**DPNAs:**: decellularized peripheral nerve allografts
**ECM:**: extracellular matrix
**FBS:**: fetal bovine serum
**HDLC:**: high-density lipoprotein cholesterol
**HE:**: hematoxylin and eosin
**HE:**: hematoxylin and eosin
**HGB:**: hemoglobin
**L/D:**: Live/Dead^®^ Cell Viability Assay
**LAM:**: laminin
**LDLC:**: low-density lipoprotein cholesterol
**LYM:**: lymphocytes,
**MCOLL:**: Myelin-collagen
**MCV:**: mean corpuscular volume
**MPV:**: mean platelet volume
**MXD:**: mixed cell count
**NAT:**: native nerves
**NEUT:**: neutrophils
**NFL:**: neurofilament
**P1:**: decellularization protocol 1
**P2:**: decellularization protocol 2
P3: decellularization protocol 3
**PAA:**: peracetic acid
**PBS:**: phosphate buffered saline
**PG:**: Proteoglycans
**PN:**: peripheral nerves
**PNF:**: peripheral nerve fibers
**PNI:**: peripheral nerve injuries
**PS:**: Picrosirius
**RBC:**: red blood cell count
**RT:**: room temperature
**SC:**: Schwann cells
**SD:**: Sondell
**SDC:**: sodium deoxycholate
**SDS:**: sodium dodecyl sulphate
**SEM:**: scanning electron microscopy
**SFI:**: Sciatic functional index
**sGAG:**: sulfatedGlycosaminoglycans
**TE:**: tissue engineering
**TEM:**: transmission electron microscopy
**TRIGL:**: triglycerides
**UREA:**: urea
**VIM:**: vimentin
**WBC:**: white blood cell count
**WST-1:**: Water-soluble tetrazolium-1
