# Supplementary material for: Comprehensive ex vivo and in vivo preclinical evaluation of novel chemo enzymatic decellularized peripheral nerve allografts
Source: Front Bioeng Biotechnol. 2023 Mar 30;11:1162684. doi: 10.3389/fbioe.2023.1162684 (PMC10111265; doi:10.3389/fbioe.2023.1162684)
Supplement: Supplementary file 1 [file Table1.DOCX]

Supplementary Material

Comprehensive ex vivo and in vivo preclinical evaluation of novel chemo enzymatic decellularized peripheral nerve allografts

**Óscar Darío García-García^1,2,3†^, Marwa El Soury^1,3†^, Fernando Campos^1^, David Sánchez-Porras^1^, Stefano Geuna^3^, Miguel Alaminos^1^, Giovanna Gambarotta^3^, Jesús Chato-Astrain^1*^, Stefania Raimondo^3 #,^ and Víctor Carriel^1#*^**

**^†^** Both authors contributed equally to this work and share first authorship.

# both authors shared the senior authorship

*** Correspondence:**

Corresponding Authors: Jesús Chato-Astrain: jchato@ugr.es; Víctor Carriel: vcarriel@ugr.es

# Supplementary Tables

Table S1: Antibodies and reagents used for immunohistochemical analyses.

| Antibody/Reagent | Dilution | Incubation | Pretreatment | Reference |
| --- | --- | --- | --- | --- |
| Rabbit Polyclonal anti- S100 antibody (Z0311) | 1: 400 | Overnight at 4 °C | Citrate buffer  pH = 6 30 min at 95 °C | DakoCytomation, Glostrup, Denmark (ref. Z0311)  RRID:AB_10013383 |
| Mouse Monoclonal Neurofilament | 1: 500 | 1 h at RT | EDTA buffer  pH = 8 25 min at 95 °C | Merck N2912, Steinheim, Germany (ref. RMdO20)  RRID:AB_477262 |
| Mouse anti-vimentin monoclonal clone V9 | 1:200 | 1 h at RT | Citrate buffer  pH = 6 25 min at 95 °C | Merck. St. Louis, MO, USA  (ref. V6630)  RRID:AB_477627 |
| Rabbit anti-laminin polyclonal | 1:200 | Overnight at 4 °C | Citrate buffer  pH = 6 25 min at 95 °C | Abcam. Cambridge, UK  (ref. ab11575)  RRID:AB_298179 |
| Rabbit monoclonal  anti-GAP-43 | 1:400 | Overnight at 4 °C | Citrate buffer  pH = 6 25 min at 95 °C | Abcam, Cambridge, UK,  (ref. ab75810)  RRID:AB_1310252 |
| ImmPRESS^®^ HRP Anti-Mouse IgG (Peroxidase) | Ready to use | 30 min at RT | - | Vector Laboratories. Burlingame, USA  (ref. MP-7402) |
| ImmPRESS^®^ HRP Anti-Rabbit IgG (Peroxidase) | Ready to use | 30 min at RT | - | Vector Laboratories. Burlingame, USA (ref. MP-7401) |
| Chromogen: Diaminobenzidine ready to use kit | - |  | - | Vector Laboratories. Burlingame, EEUU (ref. SK-4100) |
| Contrast: Harris Hematoxylin | 30 s |  | - | Thermo Scientific. Runcorn, UK  (ref. 6765004) |

Table S2: Tensile biomechanical analysis of native and decellularized nerves. Results corresponding to each biomechanical parameter are shown as mean ± standard deviation values.

|  | **Stress at fracture** | **Strain at fracture** | **Young´s Modulus** |
| --- | --- | --- | --- |
|  | **(MPa)** | **(%)** | **(MPa)** |
| **NAT** | 3.39 ± 0.70 | 55.38 ± 10.64 | 13.50 ± 3.67 |
| **Sondell** | 4.36 ± 0.46 | 55.08 ± 15.25 | 16.70 ± 5.23 |
| **P1** | 4.01 ± 0.81 | 51.75 ± 17.62 | 14.62 ± 4.76 |
| **P2** | 3.01 ± 0.49 | 39.76 ± 3.68 | 12.09 ± 2.52 |
| **P3** | 2.65 ± 0.64 | 52.16 ± 5.92 | 13.06 ± 1.87 |

**Table S3: Summary weight results of the whole leg and gastrocnemius and tibialis anterior muscles after 15 weeks of sciatic nerve repair by using DPNAs and nerve autograft techniques.** Statistically significant differences (p < 0.05) were determined with Mann–Whitney test as follows: a = vs. AUTO; b = vs SD group; c = between P1 vs. P2 groups.

|  |  | **Weight muscle morphometry** | | |
| --- | --- | --- | --- | --- |
|  |  | **Whole leg** | **Gastrocnemius** | **Tibialis anterior** |
|  |  | **(g)** | **(g)** | **(g)** |
| **AUTO** | Contralateral side | 5.87 ± 0.06 | 2.24 ± 0.05 | 0.67 ± 0.05 |
|  | Lesion side | 4.50 ± 0.16 | 1.51 ± 0.08 | 0.44 ± 0.04 |
|  | % loss | 23.31 ± 2.44 | 32.63 ± 5.00 | 34.82 ± 10.18 |
|  |  |  |  |  |
| **Sondell** | Contralateral side | 6.02 ± 0.30 | 2.48 ± 0.03 ^a^ | 0.74 ± 0.06 |
|  | Lesion side | 3.87 ± 0.37 | 1.15 ± 0.21 ^a^ | 0.36 ± 0.08 |
|  | % loss | 35.64 ± 6.89 ^a^ | 53.75 ± 8.68 ^a^ | 50.90 ± 11.78 ^a^ |
|  |  |  |  |  |
| **P1** | Contralateral side | 5.90 ± 0.33 ^c^ | 2.30 ± 0.12 ^b^ | 0.76 ± 0.03 ^a,c^ |
|  | Lesion side | 3.21 ± 0.26 ^a,b^ | 0.82 ± 0.21 ^a,b^ | 0.37 ± 0.13 |
|  | % loss | 45.44 ± 5.97 ^a^ | 63.83 ± 10.66 ^a^ | 52.01 ± 16.31 |
|  |  |  |  |  |
| **P2** | Contralateral side | 5.14 ± 0.27 ^a,b,c^ | 2.01 ± 0.36 ^b^ | 0.59 ± 0.07 ^b,c^ |
|  | Lesion side | 3.13 ± 0.53 ^a,b^ | 0.94 ± 0.22 ^a^ | 0.27 ± 0.05 ^a,b^ |
|  | % loss | 39.04 ± 10.57 ^a^ | 52.15 ± 13.13 ^a^ | 55.04 ± 3.94 ^a^ |

Table S4: Quantitative histomorphometrical analysis of nerve regeneration in the distal region of the graft.

|  | **Density** | **Total fiber number** | **Axon Diameter** | **Fiber Diameter** | **Myelin thickness** | **G-ratio** |
| --- | --- | --- | --- | --- | --- | --- |
|  | **(Nº axons·10^3^ /mm^2^)** | **(10^3^)** | **(µm)** | **(µm)** | **(µm)** |  |
| **CTR** | 12.99 ± 2.53 | 8.71 ± 0.74 | 5.33 ± 0.84 | 8.39 ± 0.82 | 1.53 ± 0.09 | 0.63 ± 0.04 |
| **AUTO** | 20.26 ± 2.57 | 16.64 ± 2.83 | 2.33 ± 0.36 | 3.50 ± 0.35 | 0.58 ± 0.05 | 0.65 ± 0.04 |
| **Sondell** | 10.27 ± 3.12 | 2.69 ± 1.61 | 2.17 ± 0.48 | 3.15 ± 0.83 | 0.49 ± 0.18 | 0.68 ± 0.02 |
| **P1** | 17.45 ± 2.30 | 8.39 ± 1.18 | 2.48 ± 0.66 | 3.42 ± 0.65 | 0.47 ± 0.04 | 0.71 ± 0.05 |
| **P2** | 22.31 ± 2.45 | 10.92 ± 4.88 | 2.11 ± 0.21 | 3.06 ± 0.20 | 0.47 ± 0.03 | 0.67 ± 0.02 |
